# The phased characteristics and countermeasures of physical exercise behavior change of rural older adults in China

**DOI:** 10.3389/fpubh.2026.1765272

**Published:** 2026-02-25

**Authors:** Wen-hao Zhang, Cong-hui He, Wen-yun Lu, Hai-pei Zhang

**Affiliations:** 1 School of Economics and Management, Shanghai University of Sport, Shanghai, China; 2 School of Physical Education, Shanghai University of Sport, Shanghai, China

**Keywords:** change rule, exercise behavior, rural older adults, stage difference, transtheoretical model

## Abstract

**Objective:**

Based on the Transtheoretical Model (TTM), this study aims to reveal the characteristics and evolutionary patterns of physical exercise behavior among rural older adults in China across different stages, providing a reference for constructing a stage-specific public sports service supply model.

**Methods:**

A cross-sectional questionnaire survey was conducted among 1,667 rural older adults in Ningde (Fujian), Shaoyang (Hunan), and Guanghan (Sichuan). Exercise behavior was classified into six stages: Precontemplation, Contemplation, Preparation, Action I (participating but irregularly), Action II (regular but less than six months), and Maintenance. Stage differences in behavioral, cognitive, environmental factors, and service demands were analyzed.

**Results:**

(1) Exercise frequency and duration increased progressively with stage advancement, but intensity declined in the Maintenance stage; activity choices expanded from low-threshold activities to structured programs. (2) Sports cognition significantly improved during the Preparation→Action I transition, but awareness of the role of exercise in chronic disease prevention remained generally weak with no stage differences. (3) “Safety” and “convenience” were foundational needs across all stages; the importance of “facilities” and “exercise guidance” became prominent during Preparation→Action I; and the role of “sports organizations” strengthened during Action I→Action II. (4) Service demands exhibited a fluctuating pattern: “sports guidance” and “sports organizations” were sensitive during Contemplation and Preparation; demand for “facilities” and “sports leaders” surged during Preparation→Action I; while demands for soft services showed no stage differences.

**Conclusion:**

Exercise behavior among rural older adults demonstrates significant stage-specific characteristics. Guidance and organizational information should be strengthened in the pre-exercise phases, facility and facilitator support ensured during the initiation phase, and diverse organizations developed during the regularization phase. Concurrently, knowledge dissemination regarding exercise interventions for chronic disease prevention should be maintained throughout all stages to address cognitive gaps.

## 1 Introduction

As of the end of 2024, China’s population aged 60 and above has reached 310 million, with a staggering 78% of older adults suffering from at least one chronic disease (1). The health challenges of the aging population have thus become a critical issue concerning national demographic quality and sustainable social development. Physical exercise has been proven as a key pathway to delaying physical function decline, reducing chronic disease risks, and enhancing quality of life (2). National policies such as the *Healthy China 2030* initiative and the *National Fitness Plan (2021–2025)* explicitly emphasize the importance of promoting scientific physical activity as a core strategy to actively address population aging. These policies advocate for the research and dissemination of sports and fitness programs and methods suitable for older adults, along with providing tailored exercise guidance and services. However, in terms of practical outcomes, the proportion of older adults who regularly engage in physical exercise remains only 33.9% (3). Policy dividends have yet to be fully translated into individual daily exercise behaviors. Particularly in rural areas, where foundational conditions are relatively weak, older adults generally face disadvantages in terms of economic income, health awareness, availability of facilities, and organizational support. Their level of participation in physical exercise and behavioral stability are significantly lower compared to their urban counterparts (4). There is thus an urgent need to deeply explore the characteristics of exercise behaviors among older adults and their related factors within the rural context.

In recent years, domestic and international research on physical exercise behavior among older adults has primarily focused on participation levels, behavioral characteristics, and influencing factors, with particular attention to urban–rural differences (5), generational disparities (6), family support (7), and the supply of public sports services (8). Overall, however, existing studies still exhibit three main shortcomings. First, they often remain at the static comparison of “whether to exercise” or “how much to exercise,” with limited exploration of the complete behavioral progression chain from the pre-intention stage to the maintenance stage. Second, most studies target urban or general older adult populations, paying insufficient attention to the stage-specific characteristics and differentiated needs of rural older adults, a disadvantaged group. Third, there is a lack of systematic explanation regarding how behavioral, cognitive, and contextual factors collectively influence “stage transitions,” making it difficult to provide practical, evidence-based support for stage-specific and targeted interventions. Based on these gaps, this study adopts the Transtheoretical Model (TTM) as its theoretical framework to conduct a stage-specific classification and empirical analysis of physical exercise behavior among older adults in typical rural regions of China. It systematically outlines the exercise behavior patterns and key influencing factors of rural older adults across different stages, identifies pain points hindering stage transitions, and proposes public sports service intervention strategies tailored to stage-specific characteristics. The study aims to achieve three innovations. First, systematically introducing the TTM into the context of physical exercise behavior among rural older adults in China, refining and deepening the stage theory of exercise behavior change within a localized context. Second, constructing an empirical mapping of “stage-behavioral characteristics-influencing factors” based on a large-sample survey to reveal the stage-specific progression patterns and key leverage points for transitions in rural older adults’ exercise behavior. Third, proposing actionable intervention pathways from a stage-matching perspective, providing decision-making references for optimizing the supply model of public sports services for rural older adults and enhancing the “targeted” effectiveness of policies.

## 2 Theoretical basis

The Transtheoretical Model (TTM) was proposed by Prochaska (9) during his study on smoking cessation among smokers. Initially applied in research on psychotherapy and behavioral change, it was later extended to the field of health behavior intervention. Sonstroem first applied the Transtheoretical Model to the study of exercise behavior (10). Building on this foundation, Prochaska and Marcus (11) systematically reviewed relevant research and developed a more comprehensive Transtheoretical Model for physical exercise. In China, research on the Transtheoretical Model for physical exercise began in 1998 (12). Guo and Yang (13) summarized the model’s core concepts, components, and research progress, noting that defining the maintenance stage solely based on the time criterion of “whether exercise has been sustained for more than 6 months” lacks sufficient objectivity and rationality, leaving room for further localization and refinement. In health behavior research, theories such as the Theory of Planned Behavior (TPB), Social Cognitive Theory (SCT), and Self-Determination Theory (SDT) are also widely used to explain physical exercise behavior. However, these theories primarily focus on psychological mechanisms such as attitudes, subjective norms, self-efficacy, or intrinsic and extrinsic motivation, tending to address questions like “whether one is willing to exercise” or “why one exercises.” In contrast, the Transtheoretical Model describes the gradual evolution of behavior over time through stages such as “precontemplation—contemplation—preparation—action—maintenance,” making it more suitable for analyzing the stage-specific characteristics and transition patterns of physical exercise behavior among rural older adults.

Regarding the internal refinement of stage structure, existing research in health behavior has found significant differences within the action and maintenance stages in terms of behavioral stability, automaticity, and relapse risk. Some scholars propose understanding behavioral change as a continuous process from “behavioral initiation” to “behavioral consolidation” and “behavioral internalization,” arguing that a single action stage is insufficient to capture the heterogeneity in the early phases of behavioral change. Additionally, habit formation theory indicates that the duration of a behavior and its regularity are two interrelated yet distinct dimensions (14). An individual may have begun a behavior but still exhibits considerable fluctuations in frequency, context, and routine, indicating that a stable habit has not yet been formed. Conversely, an individual may establish a highly regular behavioral pattern within a relatively short timeframe. Therefore, it is necessary to introduce a “behavioral regularity/stability” indicator, alongside the time threshold, to enable a more refined differentiation of the action stage.

Based on the above theoretical discussions and empirical evidence, this study adheres to the classic stage sequence of the TTM while incorporating “behavioral regularity/stability” as a supplementary dimension. This leads to the distinction between Action Stage I (already participating in physical exercise but irregularly and for less than 6 months) and Action Stage II (already engaged in a relatively regular physical exercise pattern but for less than 6 months). On one hand, this distinction aligns with the critical transition from “initiation to consolidation” in the process of habit formation, reflecting stage-specific differences in behavioral quality within the action stage. On the other hand, it is consistent with the TTM’s fundamental assumption that “stages progress toward higher stability and lower relapse risk.” This refinement enhances the model’s explanatory power in characterizing the early change processes of exercise behavior among rural older adults. It is important to note, however, that the TTM primarily centers on individual cognition and readiness as core variables. Its explanatory power is limited when addressing structural and cultural constraints, such as infrastructure availability, economic conditions, gender roles, and intergenerational care responsibilities, making it insufficient to fully encompass the complex causes of physical exercise behavior among older adults in rural contexts. These limitations will be appropriately addressed in the interpretation of results and the formulation of recommendations later in this study.

## 3 Research design

### 3.1 Questionnaire design

The stages of change constitute the core component of the transtheoretical model. The stage of Change is the core component of the Transtheoretical Model. Yin Bo (2006) provided a detailed explanation of the Transtheoretical Model as applied to physical exercise behavior, categorizing the stages of change in physical exercise into five distinct stages: precontemplation (no intention to engage in physical exercise within the next 6 months), contemplation (planning to engage in regular physical exercise within the next 6 months), preparation (planning to start physical exercise within the next 30 days and having already taken some preparatory steps), action (already participating in regular physical exercise for less than 6 months), and maintenance (having engaged in regular physical exercise for more than 6 months) (15). The classic Transtheoretical Model (TTM) defines the “Action Stage” as the phase where an individual has already begun regular physical exercise but has not sustained it for more than 6 months. This definition implies the assumption that “initiating exercise” and “forming a relatively stable routine” can be depicted using a unified time threshold. However, based on preliminary interviews and observations in this study, it was found that within the localized context of physical exercise behavior among rural older adults in China, there exists a transitional state between “initial participation” and “forming a routine.” This state is often overlooked but holds critical theoretical significance as a threshold. Many rural older adults, after initially participating in physical exercise, do not immediately develop stable exercise habits. Their exercise frequency, duration per session, and choice of activities often exhibit significant fluctuations within a week, with behavioral stability and habit strength notably weaker compared to those who have already established a regular exercise pattern. If individuals in this “initiated but not yet regularized” state are grouped together with those who “have already formed a relatively stable exercise pattern but for less than 6 months” into a single action stage, it would not only statistically obscure substantial differences in behavioral characteristics, cognitive levels, and contextual needs between the two groups but also blur the distinction between two distinct intervention objectives: “how to encourage sustained participation” and “how to help solidify habits.” This would weaken the explanatory power of the theoretical model for real-world behavioral change and reduce the precision of stage-specific interventions. Therefore, while adhering to the fundamental stage division logic of the Transtheoretical Model, this study introduces the supplementary dimension of “behavioral regularity/stability.” It further subdivides the Action Stage into action I stage (already participating in physical exercise, but irregularly and for less than 6 months) and action II stage (already participating in regular physical exercise but for less than 6 months). Consequently, the exercise behavior of older adults is classified into six stages: precontemplation stage, contemplation stage, preparation stage, action I stage, action II stage, and maintenance stage (abbreviated as PC, C, P, AI, AII, M). This refinement aims to enhance the model’s adaptability and relevance within the Chinese context and to support the investigation of exercise behavior stages among rural older adults.

Based on a review of prior research concerning the application of the Transtheoretical Model (TTM) to exercise behavior, it has been established that behavioral, cognitive, and environmental factors serve as significant determinants influencing exercise behavior change (13). Consequently, in the design of this study’s questionnaire, the investigation will be structured across these three dimensions of related factors. Additionally, to clarify the variations and characteristics in service demands across different behavioral stages, the expression of demand for public sports services is also integrated into the questionnaire content. The questionnaire content was designed across four sections: behavioral factors, cognitive factors, exercise environmental factors, and demand for public sports services (see Table 1). The assessment of behavioral factors includes exercise frequency, duration per session, exercise intensity, and types of physical activities. The assessment of cognitive factors was based on a scale developed with reference to the research outcomes of Dong et al. (16). This scale consists of 10 items encompassing dimensions such as health promotion, disease prevention, and emotional experience. Responses were collected using a 5-point Likert scale. To facilitate clearer cognitive bias analysis and simplify subsequent data processing, the responses “Strongly Agree,” “Agree,” and “Neutral” were collapsed into a single “Agree” category and assigned a score of 1. The responses “Disagree” and “Strongly Disagree” were combined into a “Disagree” category and assigned a score of 0, forming a dichotomous variable. The assessment of environmental factors for exercise was based on a scale designed with reference to the research outcomes of Zhang and Xu (17). This scale consists of 10 items covering dimensions such as fitness facilities, social interaction, exercise organization, and exercise atmosphere. Responses were measured on a 5-point Likert scale (1 = Strongly Disagree, 5 = Strongly Agree), with higher scores representing a greater perceived level of importance. The assessment of the demand of public sports service was based on a survey scale designed by referencing the specific items outlined in the National Basic Public Sports Services. The scale consists of nine items (e.g., “Is there a need to construct sports facilities suitable for older adults?”). Responses were measured on a 5-point Likert scale (1 = Strongly Disagree, 5 = Strongly Agree), with higher scores representing a stronger perceived demand.

**Table 1:** Questionnaire content table.

Variable	Question	Response options
Stage of exercise behavior	Which of the following stages best describes your current physical activity situation?	(1) Precontemplation: no intention to engage in physical exercise within the next 6 months. (2) Contemplation: planning to engage in regular physical exercise within the next 6 months. (3) Preparation: planning to start physical exercise within the next 30 days and having already taken some preparatory steps. (4) Action I: already participating in physical exercise, but irregularly and for less than 6 months. (5) Action II: already participating in regular physical exercise but for less than 6 months. (6) Maintenance: having engaged in regular physical exercise for more than 6 months.
Physical exercise habits	How often do you engage in physical exercise?	(1) Five or more times per week. (2) Three to four times per week. (3) One to two times per week. (4) One to three times per month. (5) One to two times per quarter. (6) Not sure.
	How long does each session of physical activity last?	(1) Less than 20 min. (2) 21–30 min. (3) 31–40 min. (4) 41 min-1 h. (5) Over 1 h.
	How do you feel physically after exercising (exercise intensity)?	(1) No particular sensation. (2) Slightly warm. (3) Light sweating. (4) Moderate sweating. (5) Heavy sweating
	What physical activities do you participate in? (multiple selections)	(1) Walking, climbing or running. (2) Tai Chi, Tai Chi sword, Tai Chi fan or softball exercises. (3) Aerobics, Latin dance, ballroom dancing. (4) Qigong, Baduanjin, Wujinxi. (5) Waist drum, Yangko dance, Diabolo. (6) Table tennis, badminton. (7) Basketball. (8) Specialized fitness training (e.g., strength, flexibility, balance). (9) Others.
Sports cognition	1. Regular participation in physical exercise can comprehensively improve overall health.	(1) Strongly agree (2) Agree (3) Neutral (4) Disagree (5) Strongly disagree
	2. Regular participation in physical exercise contributes to mental well-being.	
	3. Regular participation in physical exercise can extend one’s lifespan.	
	4. Regular participation in physical exercise helps prevent heart disease.	
	5. Regular participation in physical exercise leads to hypertension.	
	6. Regular participation in physical exercise leads to osteoporosis.	
	7. Regular participation in physical exercise helps to strengthen physical fitness.	
	8. Regular participation in physical exercise supports independent living.	
	9. Regular participation in physical exercise provides me with opportunities to socialize.	
	10. Regular participation in physical exercise provides a sense of personal achievement.	
Environmental factors influencing participation in physical exercise	1. Availability of dedicated sports facilities and venues.	(1) Not at all important (2) Slightly important (3) Neutral (4) Important (5) Extremely important
	2. Access to professional or qualified guidance for physical exercise.	
	3. Existence of organized groups or clubs for physical activity participation.	
	4. Availability of suitable exercise programs or personalized plans.	
	5. Ability to access desired sports services at any time.	
	6. Safety aspects associated with participating in physical exercise.	
	7. Convenience and accessibility factors for engaging in physical exercise.	
	8. Time or financial costs involved in participating in physical exercise.	
	9. Support from family.	
	10. Support from friends.	
Public sports service demands	1. Constructing sports facilities suitable for older adults.	(1) Not at all necessary (2) Slightly necessary (3) Neutral (4) Necessary (5) Extremely necessary
	2. Establishing physical exercise organizations for older adults.	
	3. Providing guidance for older adults on participation in physical exercise.	
	4. Organizing physical exercise activities suitable for older adults.	
	5. Providing health and fitness knowledge for older adults.	
	6. Offering physical fitness assessment services for older adults.	
	7. Training key facilitators for physical activity promotion among older adults.	
	8. Conducting promotion and mobilization campaigns to encourage physical exercise participation among older adults.	
	9. Strengthening institutional development for physical activity programs targeting older adults.	

### 3.2 Data collection and processing

This study employed a cross-sectional survey design. Based on multi-stage stratified sampling, rural older adults in Ningde (Fujian) in the eastern region, Shaoyang (Hunan) in the central region, and Guanghan (Sichuan) in the western region were surveyed. A total of 2,239 questionnaires were distributed. After excluding 572 invalid responses, 1,667 valid questionnaires were obtained, comprising 887 males (53.2%) and 780 females (46.8%). The distribution of participants across different stages of exercise behavior is presented in Table 2. It should be noted that the cross-sectional design does not allow for tracking the temporal evolution of exercise behavior in the same individual. Therefore, this study reveals the association patterns between exercise behavior stages and related factors at a single point in time rather than establishing strict causal relationships. Causal inferences require further validation through longitudinal studies.

**Table 2:** Distribution of the number of older adults in different exercise behavior stages.

Behavioral Stage	Number	Proportion
Precontemplation	693	41.57%
Contemplation	189	11.34%
Preparation	65	3.90%
Action I	202	12.12%
Action II	94	5.64%
Maintenance	424	25.43%

Regarding variable processing, considering the questionnaire structure and sample distribution, the sports cognition variable was dichotomously coded based on whether relevant cognition was present (0 = absent or weak cognition, 1 = present or strong cognition). This approach, on the one hand, helps avoid the issue of small cell frequencies in statistical analyses when certain response options have limited sample sizes, which could affect the stability of chi-square tests and stage comparison results. On the other hand, it facilitates clearer group comparisons across different stages of exercise behavior. It is important to note that dichotomization reduces the granularity of measurement to some extent. Therefore, in interpreting the results, the focus is primarily on the threshold effect of “whether cognition reaches a certain level,” without overextending inferences about subtle gradations in cognitive levels.

### 3.3 Validity and reliability testing

The general questions in the questionnaire underwent retest reliability testing using split-half reliability coefficients, with the average split-half reliability coefficient *r* = 0.73 (*p* < 0.01) in the *Public Sports Service Demand Scale for Older Adults Questionnaire*. This indicates that the retest reliability of the general questions in the questionnaire is good. For the reliability assessment of the questionnaire’s scales, a combined approach of Exploratory Factor Analysis (EFA) and Confirmatory Factor Analysis (CFA) was employed to evaluate measurement consistency. Results showed Cronbach’s alpha coefficients of 0.83, 0.86, and 0.89 for the scales, with KMO values of 0.74, 0.87, and 0.83, respectively. Bartlett’s sphericity test significance levels were all below 0.05. In conclusion, the *Public Sports Service Demand Scale for Older Adults* exhibits good reliability and validity based on these statistical analyses.

## 4 Results and analyses

### 4.1 Analysis of exercise behavior across different stages of change

The survey results (see Table 3) indicate significant differences (*p* < 0.001) in exercise frequency, duration, and intensity among rural older adults across various stages of change. Overall, these metrics demonstrate an evolutionary trend of “increasing from low to high, then stabilizing” as individuals progress through the stages.

**Table 3:** Characteristics of exercise behavior preferences among different types of older adults.

Exercise characteristics	Action I (N/%) *N* = 202	Action II (*N*/%) *N* = 94	Maintenance (*N*/%) *N* = 424	Sum (*N*/%) *N* = 720	F
Exercise frequency					
Three times or more per week	61 (30.1%)	56 (59.5%)	369 (87.0%)	486 (67.5%)	185.7^***^
One to two times per week	33 (16.3%)	18 (19.1%)	28 (6.6%)	79 (10.9%)	
Less than once per week	108 (53.4%)	20 (21.2%)	27 (6.3%)	155 (21.5%)	
Duration per session					
30 min or less	60 (29.7%)	23 (24.4%)	65 (15.3%)	148 (20.5%)	19.39^***^
31 min to 1 h	98 (48.5%)	41 (43.6%)	165 (38.9%)	304 (42.2%)	
Over 1 h	44 (21.8%)	30 (31.9%)	194 (45.8%)	268 (37.2%)	
Intensity per session					
No particular sensation	20 (9.9%)	3 (3.2%)	53 (12.5%)	76 (10.6%)	7.54^***^
Slightly warm	52 (25.7%)	19 (20.2%)	125 (29.5%)	196 (27.2%)	
Light sweating	70 (34.7%)	32 (34.0%)	140 (33.0%)	242 (33.6%)	
Moderate sweating	49 (24.3%)	32 (34.0%)	77 (18.2%)	158 (21.9%)	
Heavy sweating	11 (5.4%)	8 (8.5%)	29 (6.8%)	48 (6.7%)	
Exercise activities (multiple selections)					
Brisk walking, running	166 (82.2%)	75 (79.8%)	362 (85.4%)	603 (83.8%)	-
Aerobics and square dancing	21 (10.4%)	23 (24.5%)	79 (18.6%)	123 (17.1%)	
Ethnic Traditional Sports	29 (14.3%)	21 (22.3%)	41 (9.6%)	91 (12.6%)	
Small ball sports	28 (13.9%)	10 (10.6%)	33 (7.8%)	71 (9.9%)	
Large ball sports	21 (10.4%)	5 (5.3%)	7 (1.7%)	33 (4.6%)	
Specialized fitness exercises	4 (2.0%)	2 (2.1%)	13 (3.1%)	19 (2.6%)	
Other activities	17 (8.4%)	5 (5.3%)	22 (5.2%)	44 (6.1%)	

***p < 0.001.

Regarding exercise frequency, the highest number of participants (108, 53.4%) in the action I stage exercised once per week or less. In contrast, the highest proportions exercising three times per week or more were found in action II stage (59.5%) and the maintenance stage (87.0%). This suggests that the exercise behavior of rural older adults tends to evolve toward higher frequency as they progress through the stages (action I stage→action II stage→maintenance stage), meaning regular exercise habits gradually form in the later stages of exercise engagement.

For exercise duration, both action I stage and action II stage participants predominantly engaged in sessions lasting between 31 min and 1 h (48.5 and 43.6% of participants, respectively). However, older adults in the maintenance stage were more likely to exercise for 1 h or longer per session (45.8%). This demonstrates a progressive characteristic where exercise duration evolves from “moderately controlled sessions” to “actively extended durations” as individuals advance through the stages.

In terms of exercise intensity, older adults across all stages preferred low-intensity exercise characterized by “light sweating.” The peak in exercise intensity occurred in action II stage, where “moderate sweating or more” accounted for 42.5% of participants. In the maintenance stage, however, the proportion of moderate or higher intensity exercise declined to 25%, suggesting that long-term exercisers may have voluntarily reduced their exercise load or lacked ongoing scientific guidance.

In terms of exercise modality choice, “brisk walking or running” remained dominant across all three behavioral stages (approximately 80% or more). However, in action II stage and the maintenance stage, participation in organized and group-based activities such as “aerobics and square dancing” and “traditional ethnic sports” showed an increase. This indicates that as individuals progress through the stages, exercise forms gradually expand from simple, low-threshold aerobic walking to more diversified activities that incorporate social interaction and structured guidance.

In summary, the stage progression from action I stage→action II stage→maintenance stage is characterized by continuous increases in exercise frequency and duration, maintenance of moderate exercise intensity, and a shift from single to diversified exercise modalities. This aligns with the behavioral change pathway of “from trial action to stable maintenance” in the Transtheoretical Model, indicating that the results demonstrate strong theoretical explanatory power.

### 4.2 Analysis of sports cognition across different exercise behavior stages

The survey results (see Table 4) indicate that the overall sports cognition level of rural older adults tends to strengthen with progression through exercise stages, though significant differences exist across various cognitive dimensions. Overall, older adults demonstrate relatively high awareness of the general benefits of physical exercise, such as “promoting health” (SD = 0.90), “enhancing physical fitness” (SD = 0.90), and “extending longevity” (SD = 0.81), with average scores exceeding 0.8. In contrast, awareness of the role of physical exercise in preventing chronic diseases like “preventing heart disease” (SD = 0.61), “preventing hypertension” (SD = 0.57), and “preventing osteoporosis” (SD = 0.54) is generally weak, with average scores remaining around 0.5.

**Table 4:** Variance test of sports cognition in different exercise behavior stages.

Item	Promoting health	Enhancing physical fitness	Extending longevity	Improving mental health	Increasing social opportunities	Supporting independent living	Fostering a sense of achievement	Preventing heart disease	Preventing hypertension	Preventing osteoporosis
Precontemplation (PC) *N* = 693	0.85 ± 0.13	0.88 ± 0.01	0.74 ± 0.01	0.70 ± 0.01	0.67 ± 0.01	0.62 ± 0.01	0.58 ± 0.01	0.54 ± 0.01	0.52 ± 0.02	0.48 ± 0.01
Contemplation (C) *N* = 189	0.91 ± 0.02	0.92 ± 0.01	0.83 ± 0.02	0.71 ± 0.03	0.74 ± 0.03	0.68 ± 0.03	0.66 ± 0.03	0.62 ± 0.03	0.53 ± 0.03	0.52 ± 0.03
Preparation (P) *N* = 65	0.84 ± 0.04	0.72 ± 0.05	0.73 ± 0.05	0.67 ± 0.05	0.66 ± 0.05	0.63 ± 0.06	0.52 ± 0.06	0.55 ± 0.06	0.61 ± 0.06	0.63 ± 0.06
Action I (AI) *N* = 202	0.94 ± 0.01	0.92 ± 0.01	0.88 ± 0.02	0.86 ± 0.02	0.85 ± 0.02	0.81 ± 0.02	0.77 ± 0.02	0.67 ± 0.03	0.62 ± 0.03	0.59 ± 0.03
Action II (AII) *N* = 94	0.96 ± 0.01	0.92 ± 0.02	0.85 ± 0.03	0.79 ± 0.04	0.87 ± 0.02	0.78 ± 0.04	0.76 ± 0.04	0.63 ± 0.04	0.67 ± 0.04	0.61 ± 0.05
Maintenance (M) *N* = 424	0.96 ± 0.01	0.93 ± 0.01	0.88 ± 0.01	0.89 ± 0.01	0.84 ± 0.01	0.83 ± 0.01	0.84 ± 0.01	0.68 ± 0.02	0.62 ± 0.02	0.60 ± 0.02
Total *N* = 1,667	0.90 ± 0.01	0.90 ± 0.01	0.81 ± 0.01	0.77 ± 0.01	0.76 ± 0.01	0.71 ± 0.01	0.69 ± 0.01	0.61 ± 0.01	0.57 ± 0.01	0.54 ± 0.01
F	10.12^***^	6.67 ^***^	9.51 ^***^	15.26 ^***^	12.75^***^	14.98 ^***^	20.79^***^	5.17 ^***^	6.67^***^	3.64 ^***^
PC-C	−0.062^***^	−0.036	−0.092^***^	−0.015	−0.066	−0.06	−0.072^**^	−0.076	−0.009	−0.036
C-P	0.069	0.197 ^***^	0.097	0.042	0.084	0.057	0.138^**^	0.07	−0.08	−0.106
P-AI	−0.099^**^	−0.202 ^***^	−0.147 ^***^	−0.189 ^***^	−0.194 ^***^	−0.186 ^***^	−0.254^***^	−0.119	−0.008	0.036
AI- AII	−0.022	−0.004	0.035	0.068	−0.015	0.029	0.011	0.034	−0.046	−0.022
AII-M	0.005	−0.008	−0.031	−0.098 ^**^	0.028	−0.047	−0.076	−0.045	0.047	0.008

*p < 0.05, **p < 0.01, ***p < 0.001.

According to analysis of variance and inter-stage comparisons, significant differences exist across all 10 sub-items of sports cognition at different stages of exercise behavior (*p* < 0.001). Notable improvements in multiple cognitive aspects—such as “promoting health,” “improving mental health,” “increasing social opportunities,” and “fostering a sense of achievement”—primarily occur during the transition from the “preparation stage” to “action I stage.” This suggests that the critical behavioral initiation point from “intending to exercise” to “starting to exercise” serves as an important window for a leap in sports cognition levels.

While overall cognition shows an upward trend, scores for awareness of “preventing heart disease,” “preventing hypertension,” and “preventing osteoporosis” remain consistently low across all stages (approximately 0.5–0.7 points). More importantly, no significant differences were observed in these three cognitive areas between any two adjacent exercise stages. This indicates a “structural gap” in rural older adults’ awareness of the role of physical exercise in preventing chronic diseases, and this cognitive shortcoming is not naturally addressed as their exercise experience accumulates.

During the transition from “action I stage” to subsequent stages (action II stage, maintenance stage), the extent of improvement in most cognitive items diminishes, and differences become less significant. This suggests that once older adults begin exercising and enter the action stage, their foundational sports cognition is largely formed, with subsequent stages showing evident cognitive consolidation.

In summary, the development of sports cognition among rural older adults exhibits characteristics of imbalance and stage-specific thresholds. On one hand, general health awareness improves with exercise participation, achieving critical breakthroughs during the behavioral initiation phase (preparation stage→action I stage). On the other hand, awareness of the role of exercise in preventing chronic diseases remains persistently low, representing a prominent gap in the cognitive dimension. These findings suggest that generalized sports promotion has limited impact on enhancing cognition among older adults who are already exercising. Instead, targeted education on specialized knowledge, such as chronic disease prevention, should become a key intervention focus across all stages—particularly for those who have not yet started exercising or are in the early stages of participation.

### 4.3 Analysis of related factors across different exercise behavior stages

As shown in Table 5, the factors “safety of participation” (SD = 4.30), “convenience of participation” (SD = 4.02), and “availability of dedicated sports facilities” (SD = 3.99) had average scores around 4.0. These are the three most prominent concerns for older adults across all stages when participating in physical exercise, serving as foundational guarantees for exercise engagement, and their importance does not vary significantly across stages. In contrast, “support from friends” (SD = 3.17) was the least correlated factor. Within the specific socio-cultural context and life realities of rural China, the daily activity circles, decision-making dependencies, and emotional support of older adults primarily revolve around the family. Social interactions with peers may be more centered on casual conversation rather than participating in organized physical exercise together, which explains why the average score for “support from family” (SD = 3.68) is notably higher than that for “support from friends” (SD = 3.17).

**Table 5:** Variance test of related factors in different exercise behavior stages.

Item	Safety of participation	Convenience of participation	Availability of dedicated sports facilities	Cost of exercise participation	Availability of exercise guidance	Availability of suitable exercise programs	Ease of access to sports services	Support from family	Participation in sports organizations	Support from friends
Precontemplation (PC) *N* = 693	4.29 ± 0.35	4.01 ± 0.36	3.91 ± 0.40	3.93 ± 0.38	3.64 ± 0.43	3.64 ± 0.41	3.57 ± 0.42	3.78 ± 0.44	3.47 ± 0.42	3.08 ± 0.41
Contemplation (C) *N* = 189	4.29 ± 0.58	4.05 ± 0.62	4.07 ± 0.72	3.78 ± 0.72	3.96 ± 0.76	3.90 ± 0.68	3.84 ± 0.80	3.50 ± 0.83	3.79 ± 0.71	3.13 ± 0.78
Preparation (P) *N* = 65	4.06 ± 0.11	3.89 ± 0.12	3.62 ± 0.14	3.63 ± 0.12	3.42 ± 0.15	3.74 ± 0.13	3.65 ± 0.13	3.42 ± 0.13	3.51 ± 0.14	2.98 ± 0.13
Action I (AI) *N* = 202	4.37 ± 0.06	4.04 ± 0.06	4.10 ± 0.07	3.90 ± 0.06	3.80 ± 0.07	3.85 ± 0.07	3.82 ± 0.07	3.60 ± 0.08	3.67 ± 0.07	3.24 ± 0.07
Action II (AII) *N* = 94	4.21 ± 0.09	3.86 ± 0.10	4.13 ± 0.11	3.77 ± 0.10	4.00 ± 0.10	3.91 ± 0.10	3.90 ± 0.10	3.56 ± 0.12	3.99 ± 0.10	3.35 ± 0.11
Maintenance (M) *N* = 424	4.35 ± 0.03	4.09 ± 0.04	4.06 ± 0.05	3.78 ± 0.04	3.87 ± 0.05	3.83 ± 0.05	3.88 ± 0.05	3.70 ± 0.05	3.78 ± 0.05	3.30 ± 0.05
Total *N* = 1,667	4.30 ± 0.02	4.02 ± 0.02	3.99 ± 0.03	3.85 ± 0.02	3.77 ± 0.03	3.76 ± 0.03	3.73 ± 0.03	3.68 ± 0.02	3.64 ± 0.03	3.17 ± 0.02
F	1.73	1.39	3.97***	2.28*	5.65***	3.47***	5.78***	3.06***	7.92***	3.11***
PC-C	−0.003	−0.04	−0.166	0.149	−0.314^***^	−0.263 ^***^	−0.267 ^***^	0.286^***^	−0.328 ^***^	−0.045
C-P	0.229	0.155	0.459 ^***^	0.147	0.542^***^	0.166	0.195	0.082	0.286	0.142
P-AI	−0.305	−0.147	−0.489 ^***^	−0.265	−0.382 ^***^	−0.108	−0.171	−0.184	−0.166	−0.253
AI- AII	0.154	0.178	0.024	0.13	−0.203	−0.068	−0.087	0.035	0.316^**^	−0.113
AII-M	−0.141	−0.228	0.066	−0.017	0.13	0.085	0.022	−0.132	0.211	−0.049

*p < 0.05, **p < 0.01, ***p < 0.001.

The results of the analysis of variance indicate that there were no significant differences across adjacent stages for “safety of participation,” “convenience of participation,” “cost of exercise participation,” and “support from friends.” This suggests that safety and convenience are fundamental needs throughout the entire process of exercise decision-making among older adults and do not change with variations in exercise stage. The study found that significant differences in the ratings for the factors “availability of dedicated sports facilities” and “availability of exercise guidance” emerged between the “preparation stage” and “action I stage” (P_P-AI_ < 0.001). This indicates that access to facilities and professional guidance is highly correlated with the critical transition point for older adults from “intending to exercise” to “starting to exercise.” With the exception of the factors mentioned above, significant differences in the remaining factors were primarily observed between the “precontemplation stage” and “contemplation stage.” This suggests that the emergence of the intention to engage in exercise behavior is the most “sensitive window period” during which environmental factors exert their strongest influence.

In summary, a dynamic association pattern exists between environmental factors and the exercise behavior stages of rural older adults. During the behavioral initiation phase (precontemplation stage→contemplation stage), creating a comprehensive supportive atmosphere for older adults is essential. In the behavioral activation phase (preparation stage→action I stage), providing facilities and professional guidance to older adults becomes critical. In the behavioral regularization phase (action I stage→action II stage), the role of sports organizations becomes more prominent. This provides clear empirical evidence for implementing stage-specific and priority-focused environmental interventions.

### 4.4 Analysis of public sports service needs across different exercise behavior stages

As shown in Table 6, the data indicate that rural older adults have relatively strong desires for all aspects of public sports services, with average need scores ranging from 3.97 to 4.42. Specifically, the demand levels for “physical fitness monitoring” (SD = 4.42), “sports facilities” (SD = 4.41), and “sports and fitness knowledge” (SD = 4.34) were relatively high. In contrast, the demand levels for “institutional development” (SD = 4.13), “sports promotion and mobilization” (SD = 4.06), and “training of key facilitators” (SD = 3.97) were relatively lower. Overall, the evolution of public sports service demand across stages is not characterized by linear progression. A notable decline in multiple service demands was observed in the “preparation stage,” forming a demand trough. This may be attributed to the fact that older adults in this stage possess the strongest intrinsic motivation and have higher confidence in overcoming initial barriers, thereby temporarily reducing their explicit reliance on certain types of external support.

**Table 6:** Variance test of exercise demand in different exercise behavior stages.

Item	Physical fitness monitoring	Sports facilities	Sports and fitness knowledge	Physical activity	Sports guidance	Sports organizations	Institutional development	Sports promotion and mobilization	Sports leaders
Precontemplation (PC) *N* = 693	4.42 ± 0.04	4.39 ± 0.05	4.32 ± 0.04	4.22 ± 0.04	4.11 ± 0.05	4.05 ± 0.05	4.05 ± 0.05	3.97 ± 0.05	3.83 ± 0.05
Contemplation (C) *N* = 189	4.57 ± 0.06	4.51 ± 0.05	4.43 ± 0.07	4.36 ± 0.06	4.37 ± 0.06	4.23 ± 0.06	4.16 ± 0.07	4.03 ± 0.07	3.89 ± 0.08
Preparation (P) *N* = 65	4.06 ± 0.13	4.02 ± 0.12	4.14 ± 0.11	4.04 ± 0.12	4.00 ± 0.13	4.02 ± 0.11	4.20 ± 0.12	4.10 ± 0.13	3.73 ± 0.15
Action I (AI) *N* = 202	4.32 ± 0.07	4.45 ± 0.06	4.34 ± 0.06	4.22 ± 0.07	4.20 ± 0.07	4.15 ± 0.07	4.15 ± 0.07	4.12 ± 0.07	4.06 ± 0.07
Action II (AII) *N* = 94	4.52 ± 0.08	4.25 ± 0.10	4.38 ± 0.09	4.41 ± 0.08	4.37 ± 0.09	4.32 ± 0.08	4.19 ± 0.10	4.12 ± 0.08	4.20 ± 0.10
Maintenance (M) *N* = 424	4.42 ± 0.05	4.46 ± 0.04	4.35 ± 0.05	4.31 ± 0.05	4.28 ± 0.05	4.30 ± 0.05	4.17 ± 0.05	4.11 ± 0.05	4.08 ± 0.05
Total *N* = 1,667	4.42 ± 0.03	4.41 ± 0.02	4.34 ± 0.03	4.27 ± 0.03	4.22 ± 0.03	4.17 ± 0.03	4.13 ± 0.03	4.06 ± 0.03	3.97 ± 0.03
F	3.41***	3.39 ***	0.97	2.04^*^	3.09***	3.48***	0.82	1.18	3.93***
PC-C	−0.146	−0.125	−0.11	−0.14	−0.254^***^	−0.180^**^	−0.114	−0.063	−0.06
C-P	0.509 ^***^	0.494 ^***^	0.278	0.318^**^	0.366^**^	0.205	−0.042	−0.074	0.16
P-AI	−0.255	−0.430 ^***^	−0.196	−0.176	−0.199	−0.132	0.058	−0.015	−0.324^**^
AI- AII	−0.203	0.23	0.044	0.191	−0.172	−0.169	−0.039	−0.006	−0.139
AII-M	0.097	−0.211^**^	0.033	0.094	0.09	0.026	0.013	0.009	0.113

*p < 0.05, **p < 0.01, ***p < 0.001.

Significant changes in the demand scores for “sports guidance” and “sports organizations” primarily occurred between the “precontemplation stage” and the “contemplation stage.” This reveals that, when the intention to exercise first emerges, the expectation for professional guidance and socialized platforms becomes prominent. These services are psychologically perceived as key “catalysts” for lowering the entry barrier and increasing the credibility of exercise.

Significant surges in the demand scores for “sports facilities” (P_P-AI_<0.001) and “sports leaders” (P_P-AI_<0.01) occurred during the transition from the “preparation stage” to the “action I stage” (e.g., significant stage difference P-AI for the demand of “sports facilities”). This indicates that, during the critical shift from “intending to exercise” to “starting to exercise,” the urgent need for tangible support (facilities) and local facilitators (leaders) is instantly activated. These represent the most direct foundational conditions supporting the first step into exercise.

In comparisons between adjacent stages (PC-C, C-P, P-AI, AI-AII, AII-M), the demands for “sports and fitness knowledge,” “institutional development,” and “sports promotion and mobilization” showed no significant differences across any adjacent pairs. This suggests that rural older adults lack a clear, differentiated perception of these “soft” service systems related to knowledge dissemination, institutional safeguards, and promotional efforts. This may stem from the chronic absence or superficial implementation of such services in rural areas.

In summary, public service provision must shift from a “one-size-fits-all” approach to “stage-adapted” delivery. During the pre-exercise phases (contemplation stage, preparation stage), the focus should be on providing professional guidance and information about sports organizations to facilitate the conversion of intention into action. During the behavioral initiation phase (preparation stage→action I stage), it is essential to ensure the availability of sports facilities and the presence of community sports leaders to support older adults in commencing exercise. Meanwhile, strengthening “soft” services—such as sports knowledge dissemination, institutional development, and promotion—constitutes a foundational, cross-stage effort required to address the current cognitive gap among rural older adults.

## 5 Discussion

### 5.1 Characteristics of exercise behavior among rural older adults across different exercise behavior stages

In previous studies on the relationship between exercise stages and exercise levels, Wilson et al. (18) found that individuals at higher stages of exercise behavior typically report higher levels of exercise engagement. The results of this study also show a similar correlation pattern: the exercise frequency and duration per session among rural older adults generally increase as their behavioral stages progress. This cross-sectional finding aligns with the direction of Wilson’s conclusion. However, after entering the maintenance stage, the self-reported exercise intensity of the older adults in the sample did not continue to rise; instead, it showed a certain degree of decline, with more individuals preferring moderate-to-low intensity levels, such as “light sweating.” This phenomenon may be related to long-term exercisers voluntarily reducing their exercise load for self-protection, the combined effects of chronic conditions and changes in physical capacity, or the long-term lack of systematic, continuous exercise guidance tailored to different stages in rural areas. The specific underlying mechanisms require further investigation in subsequent studies. The recently published *Scientific Exercise Guidelines for Older Adults (2023)* also addresses the staged nature of exercise behavior in older adults and emphasizes the principle of progressive overload. However, it does not provide specific answers to questions such as how to progress appropriately or what considerations should be taken at different stages. The patterns of intensity variation and exercise modality choices observed in this study provide some evidence for the practical necessity of strengthening stage-specific exercise guidance and safety management in rural contexts. Regarding exercise modality choices, this study found that walking and jogging are the primary forms of participation for rural older adults, with activity choices across all stages highly concentrated on a few low-barrier aerobic options. This aligns with the findings of scholars such as Ding et al. (19), Chen (20), and Li and Wang (21), who observed that older adults generally prefer low-intensity aerobic activities (e.g., brisk walking, jogging, square dancing). This concentrated activity structure reflects the practical constraints faced by older adults in terms of health status, exercise experience, and risk tolerance. On the other hand, it also suggests that the current public sports provision in rural areas remains limited in terms of activity variety, organizational forms, and professional guidance, leaving relatively little room for older adults to explore and choose independently.

Based on the behavioral characteristics and activity preference patterns described above, it is necessary at the policy and practice levels to more closely align existing fitness guidelines and exercise prescription systems with the exercise behavior stages of rural older adults. First, in the development of guidelines and prescriptions, the stage classifications of the Transtheoretical Model can be incorporated to provide tiered, actionable recommendations for exercise frequency, duration, intensity, and modality types at different stages. In particular, for individuals in the Action and Maintenance stages, specific guidance on how to reasonably control exercise loads and avoid overexertion or premature withdrawal while ensuring safety should be provided. This would address the “post-peak decline in intensity” phenomenon observed in this study. Second, in the areas of promotion and education, stage-specific guidance should be disseminated to rural communities through grassroots health service institutions, township (street-level) sports organizations, and village-level activity platforms. This would improve the accessibility and comprehensibility of guideline content for older adults and reduce behavioral deviations caused by information asymmetry. Third, in terms of implementation and service provision, while continuing to ensure access to low-barrier activities like walking, diversified activity types and organizational forms that match the needs of different stages should be systematically introduced. For example, entry-level and progressively adjustable activity combinations could be offered for individuals in the Preparation and Action stages, while more varied group-based and socially engaging exercise options could be provided for those in the Maintenance stage.

### 5.2 Characteristics of sports cognition among rural older adults across different exercise behavior stages

Sports cognition serves not only as a prerequisite for physical activity behavior but also as a key factor influencing an individual’s adherence to and sustainability of exercise routines. However, this cognitive capacity is not static throughout the behavioral change process. Gao (22) found that as individuals progress from lower to higher stages of exercise behavior, their perceived benefits of exercise gradually strengthen, and their intrinsic motivation correspondingly increases. This study similarly identified significant differences in the sports cognition of rural older adults across different stages, with a clear trend of enhancement observed in the stage distribution. The distinctive finding, however, is that significant differences do not exist between all adjacent stages. Significant differences between neighboring stages are concentrated specifically between the preparation stage and action I stage, while few significant differences are observed from action I stage through the maintenance stage. In other words, within this sample, the differences in sports cognition levels are more pronounced between the early stages of “not yet taking action” (precontemplation stage, contemplation stage and preparation stage) and the stages of “already exercising.” In contrast, cognition tends to stabilize at a higher level among the stages where individuals are “already active.” The relevant results indicate: a stronger association exists between higher sports cognition levels and “whether exercise has been initiated” (i.e., crossing the threshold from preparation to action I stage), whereas the relationship between cognition and the long-term maintenance of exercise behavior is not as linearly strong as expected. It is important to emphasize that cross-sectional data cannot determine whether “increased cognition prompts individuals to enter a higher stage” or “individuals who have already entered a higher stage develop increased cognition through exercise experience and information exposure”—both directions are plausible. Furthermore, rural older adults with higher education levels, better health literacy, adequate family support, and higher income exhibit significant stage-specific differences in sports cognition and exercise behavior stages. This is particularly evident at the critical transition point from the Preparation stage to the initial action stage, where a higher consistency between the two is observed. The results further reveal that rural older adults possess relatively high levels of cognition regarding the benefits of exercise for “promoting health,” “enhancing physical fitness,” and “extending longevity.” This aligns with the trend observed in earlier research by Zhai et al. (23) and Zheng and Jiang (24), where older adults gradually shift from viewing exercise as “useless” to “valuable,” indicating that the macro-level cognition of “exercise is beneficial” has gained some foundation among rural older adults. In contrast, cognition related to preventing specific chronic diseases such as hypertension, heart disease, and osteoporosis remains at a notably low level, with no significant differences observed between adjacent stages. This indicates a widespread, cross-stage cognitive gap among rural older adults regarding the role of exercise in chronic disease prevention. Notably, the *Opinions on Building a Higher-Level Public Service System for Fitness and Physical Activity* explicitly advocates for promoting exercise intervention programs and methods for common chronic diseases and champions the concept that “exercise is medicine” (25). The findings of this study empirically reveal a gap between policy advocacy and the actual cognition of older adults at the grassroots level, particularly in the dissemination of knowledge about exercise interventions for chronic diseases.

Based on the observed correlation patterns, two targeted practical implications can be proposed. First, education and advocacy regarding the role and value of exercise in preventing and managing chronic diseases should be prioritized as a key task to address the “cross-stage common cognitive gap” among rural older adults, rather than merely reinforcing existing awareness of “general health benefits.” Second, given the finding that cognitive differences are more pronounced between the preparation stage and action I stage, sports knowledge dissemination efforts at the rural level should moderately shift their focus forward. Priority should be given to covering populations in the precontemplation, contemplation, and preparation stages who have not yet established stable exercise behaviors. Through channels such as grassroots healthcare institutions, family doctor contract services, and village-level cultural and sports platforms, easily understandable and highly relevant exercise and health information should be continuously provided to these groups.

### 5.3 Differences in the impact of environmental factors on rural older adults across different exercise behavior stages

Previous research exploring factors influencing exercise behavior change has predominantly focused on the interaction between personal factors—including demographic characteristics, physical and mental health status—and exercise behavior change. Studies by Zhang et al. (26), Xu et al. (27, 28), Jiménez-Zazo et al. (29), Alves et al. (30), and Picorelli et al. (31), among others, have found that personal-level factors such as age, gender, marital status, education level, and economic income have direct or indirect effects on exercise behavior change in older adults. While significant progress has been made in this area, research specifically examining the relationship between exercise stages and contextual environmental factors—including exercise environment and social support—remains relatively limited. van Stralen et al. (32) identified that environmental factors such as social support and perceived facility accessibility influence the exercise stages of older adults, and these factors vary in their impact across different stages, providing important insights into the “environment-stage” relationship.

This study examined the subjectively perceived exercise environment of rural older adults across multiple dimensions—including safety of participation, convenience of participation, availability of dedicated facilities, access to exercise guidance, participation in sports organizations, and support from family and friends—and compared these perceptions with their exercise behavior stages. The results indicate that the average scores for “safety of participation” and “convenience of participation” were relatively high across all stages, with no significant differences between adjacent stages. This suggests that safety and convenience represent universal concerns for rural older adults at different stages, rather than factors that distinctly differentiate stages. In contrast, the scores for items such as “availability of dedicated sports facilities” and “availability of exercise guidance” showed a more noticeable increase during the transition from the Preparation stage to action I stage, while the importance rating for “participation in sports organizations” increased during the transition from action I stage to action II stage. This indicates that age-friendly facilities and professional guidance are more prominently associated with “whether exercise is initiated,” whereas organizational participation is more strongly linked to “the progression from initial action toward stable and regularized behavior.” Further analysis revealed that opportunities for participating in sports organizations, perceiving facility convenience, and accessing professional guidance are themselves influenced by a combination of structural factors such as health status, income level, transportation conditions, and family caregiving responsibilities. For example, older adults with better health, relatively more favorable economic conditions, and residence closer to village or community activity centers are more likely to frequently use sports facilities, receive professional guidance, and participate in sports organizations, while also being more capable of maintaining higher exercise frequency and regularity. Under a cross-sectional design, these structural advantages may manifest both as “higher environmental scores” and as “being in a higher stage,” potentially amplifying the observed association between environmental factors and behavior stages.

Based on this, policy and practical implications aligned with the correlation patterns observed in this study are proposed. First, given that “safety of participation” and “convenience of participation” are consistently high across stages with limited differences, they can be regarded as “baseline conditions” for rural older adults’ participation in exercise. In practice, measures should be taken to alleviate concerns about safety risks by improving safety standards for rural sports facilities, strengthening routine maintenance and accountability, and optimizing the layout of rural roads and sports venues. Additionally, enhancing temporal and spatial accessibility of exercise should be achieved by locating facilities nearby and arranging reasonable operating hours. Second, considering that “availability of dedicated sports facilities” and “availability of exercise guidance” demonstrated stronger stage differentiation during the transition from the Preparation stage to action I stage, when designing exercise intervention programs for rural older adults, targeted support in these two aspects could be moderately increased for individuals in the precontemplation, contemplation, and preparation stages. Examples include installing age-appropriate equipment in village or community activity spaces and offering regular introductory guidance courses to provide more suitable environmental support for the critical transition from “considering exercise” to “initiating action.” Third, for older adults already in action I stage or beyond, particularly those progressing toward action II stage and the maintenance stage, emphasis could be placed on developing diverse sports organizations such as senior sports associations, morning exercise groups, and square dancing teams. This would enhance their social connections and exercise norms, gradually integrating exercise into their daily routines. These recommendations do not imply that environmental interventions will inevitably lead to stage transitions. Instead, they aim to align the direction of public sports service environment development more closely with the stage-environment correlation patterns observed in this study, thereby improving the targeting and potential effectiveness of intervention resource allocation.

### 5.4 Characteristics of public sports service demand among rural older adults across different stages

An analysis of variations in public sports service demand across different stages reveals that significant differences exist only for certain types of services. No significant differences were observed in the demand for “sports and fitness knowledge,” “institutional development,” or “sports promotion and mobilization.” This suggests a relatively low awareness and perception of these “soft” public sports services among rural older adults in China, indicating a persistent structural imbalance in rural public sports service provision that prioritizes “hardware” (facilities) over “software” (knowledge, systems, and so on) (33). On one hand, tangible services such as venues and facilities are more easily perceived and evaluated; on the other hand, soft services related to systems and governance at the grassroots level often exist in relatively implicit forms. Older adults have limited awareness of their content and functions, making it difficult for clear stage-specific differences to emerge in questionnaire responses. Concurrently, the distribution of public sports service demands among rural older adults across different stages does not follow a linear increase but rather exhibits a differentiated pattern of “slightly higher in early stages—relatively lower in the preparation stage—rising again in action stages.” Specifically, the overall demand level in the Preparation Stage is relatively the lowest among all stages, indicating that older adults in this stage already possess strong intrinsic behavioral motivation and are more inclined to rely on their own initiative to attempt exercise, showing a reduced subjective dependence on external services compared to those in the precontemplation and contemplation stages. However, once they actually engage in exercise practice, older adults in action I stage, action II stage, and the maintenance stage tend to develop heightened expectations for “higher-level services.” Nevertheless, this pattern may also be influenced by other factors, such as differences between the Preparation Stage sample and other stages in terms of health status, prior service usage experiences, and trust expectations regarding government services. As this study did not systematically control for these potential confounding factors, the observed stage-specific differences in service demand are more appropriately interpreted as “cross-sectional differences in public service expectations among groups at different stages,” rather than direct evidence that “changes in service provision lead to individual stage progression.”

Additionally, in comparisons between adjacent stages, this study found that significant differences in public sports service demands are primarily concentrated during the transition from the contemplation stage to the preparation stage and from the preparation stage to action I stage. On one hand, the demand scores for “sports guidance” and “sports organizations” showed an increasing trend during the transitions from precontemplation stage to contemplation stage and from contemplation stage to preparation stage. This suggests that in the early stages before stable exercise behavior is formed, older adults are more sensitive to expectations of “having someone to teach” and “having a group to join.” On the other hand, the demand for “sports facilities” and “sports leaders” increased more noticeably during the transition from the preparation stage to action I stage, indicating that when older adults are ready to begin actual exercise, suitable physical spaces and key facilitators become more prominent in their subjective demand structure. In contrast, between action I stage and the maintenance stage, most service items showed no significant differences in demand, reflecting that once exercise behavior is integrated into daily routines, the role of public sports services shifts more toward maintenance support rather than acting as a triggering threshold. It is important to note that these “stage-sensitive windows” may also intersect with differences in health status, income levels, and prior service usage experiences among older adults at different stages. Therefore, caution should be exercised when translating these findings into intervention strategies.

Based on this, practical implications aligned with the correlation patterns observed in this study are proposed. First, while continuing to emphasize the construction of tangible infrastructure such as venues and facilities, deliberate efforts should be made to increase the supply of soft public sports services, particularly in areas like sports knowledge dissemination, institutional improvement, and sports promotion and mobilization. Enhancing the visibility and comprehensibility of soft service content for rural older adults will enable them to more clearly perceive the potential support of institutional arrangements, regulatory designs, and educational approaches for their exercise behavior, which is foundational for optimizing the effectiveness of rural sports governance. Second, in public sports service reforms, resource allocation could be moderately shifted forward to focus more on rural older adults in the pre-exercise stages (precontemplation, contemplation and preparation stage). This should particularly involve service items that show higher sensitivity in adjacent stage comparisons, such as “sports guidance,” “sports organizations,” “sports facilities,” and “sports leaders,” to explore service combinations tailored to stage-specific characteristics. For example, in the precontemplation and contemplation stages, efforts could focus on enhancing clarity about exercise pathways through sports guidance and information about sports organizations. During the transition from preparation to action I stage, priority could be given to ensuring the availability of age-friendly venues and facilitator teams to provide more direct support for “taking the first step.” Third, digital tools could be moderately introduced to promote the digital transformation of rural public sports services, establishing more convenient channels for demand expression and service matching mechanisms. This would enable rural older adults at different stages to more clearly articulate their specific needs for guidance, organizations, facilities, and facilitators through mobile terminals, village-level service platforms, and other means.

## 6 Conclusion

Faced with the deepening trend of population aging and the high prevalence of chronic diseases, addressing the health challenges of older adults has become a major priority for China in the new era. Investigating the exercise behaviors of rural older adults through a staged approach aligns with the strategic needs of both the health and sports sectors in China, while also facilitating a shift among rural older adults from “passive health management” to “active health engagement.” Since exercise behavior change is not static but a gradual and dynamic process, a deeper understanding of the progression of exercise behavior change among rural older adults is essential. This requires thoughtful consideration of how to guide older adults toward active exercise participation and habit formation. This study conducted a multidimensional analysis and comparative assessment of exercise behaviors, related factors, and demands among rural older adults across different stages. It elucidated the behavioral characteristics and challenges faced by older adults at various stages and proposed targeted solutions at both national and local levels. The aim is to contribute to strategies for guiding older adults toward scientifically sound exercise practices and fostering proactive health habits.

However, this study has several limitations in its research design and methodology: first, the study employed a cross-sectional questionnaire survey. Cross-stage comparisons reflect differences among groups at different stages at a single point in time, rather than the actual transition process of the same individuals over time. Therefore, some dynamic interpretations should be understood as descriptive inferences. Second, the sample was drawn from specific rural areas, including Ningde (Fujian), Shaoyang (Hunan), and Guanghan (Sichuan). Variations in regional culture, economic development levels, and public service foundations mean that the conclusions have certain boundaries in terms of external validity and generalizability. It is not straightforward to extrapolate the findings to all rural older adults nationwide. Third, this study used the Transtheoretical Model as the primary analytical framework to depict the stages of exercise behavior among rural older adults. However, the Transtheoretical Model itself centers on individual cognition and behavioral readiness. Its explanatory power regarding structural and cultural constraints—such as infrastructure provision, economic conditions, gender roles, and family care responsibilities—is limited. Consequently, it may not fully encompass the complex causes of exercise behavior among older adults in rural contexts.

Future research could expand upon this study in the following directions: first, adopt longitudinal tracking or cohort study designs, incorporating objective data such as accelerometer readings, wearable device data, and public sports service usage records. This would allow for mapping the dynamic trajectories of exercise behavior stage transitions among rural older adults and testing the applicability of the Transtheoretical Model’s stage classification and transition pathways within the local context, as well as identifying potential revisions. Second, introduce multi-level and multivariate statistical models to more systematically control for key confounding factors such as age, gender, health status, income, and family structure. This would enable comparisons of the relative and independent contributions of behavioral, cognitive, environmental, and service demand factors across different levels. Simultaneously, excessive simplification in scale processing should be avoided to enhance measurement sensitivity and explanatory power. Third, theoretically, integrate the Transtheoretical Model with frameworks such as the Theory of Planned Behavior, Social Cognitive Theory, and Self-Determination Theory. Conduct comparative studies across broader regions, different family structures, gender groups, and intergenerational populations. Use experimental or quasi-experimental methods to test the actual effectiveness of “stage-specific interventions.” Through the interaction of theoretical deepening, methodological refinement, and policy practice, this approach would provide more robust evidence and practical pathways to support the cultivation of scientifically sound fitness practices and proactive health behaviors among rural older adults.

## Data Availability

The original contributions presented in the study are included in the article/supplementary material, further inquiries can be directed to the corresponding author.
